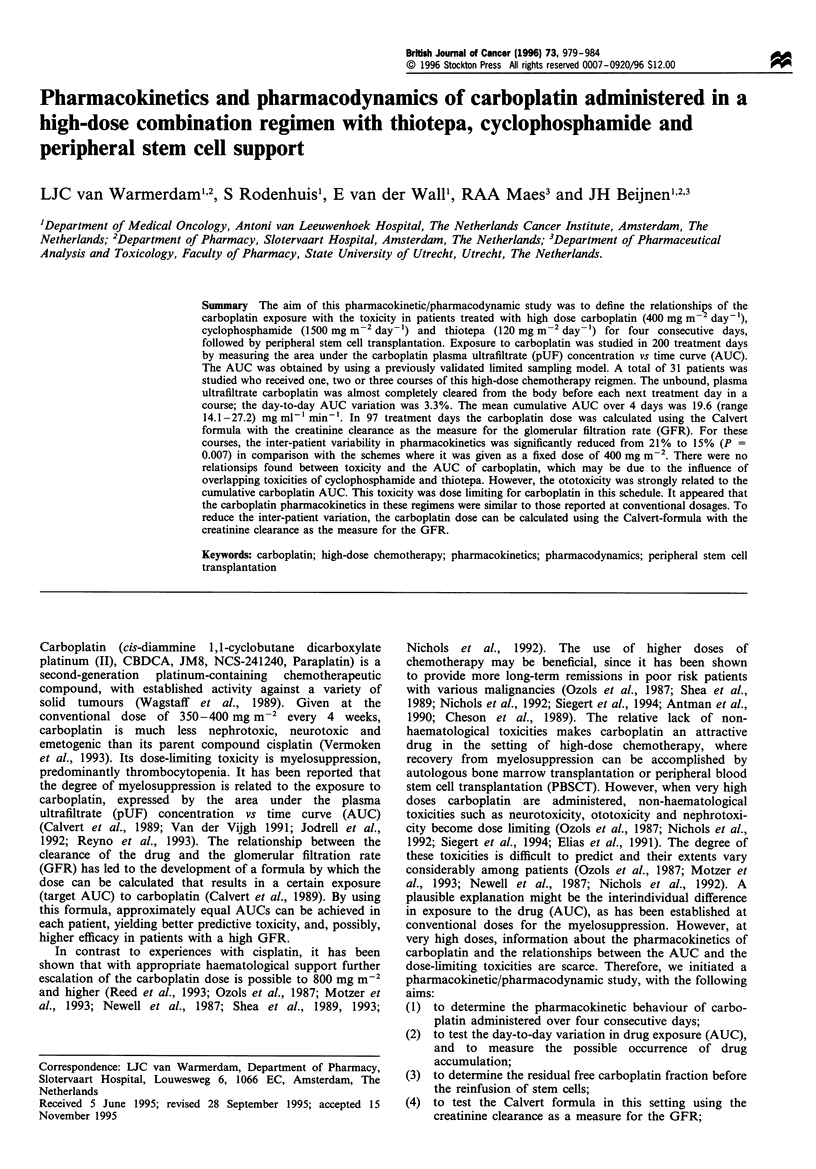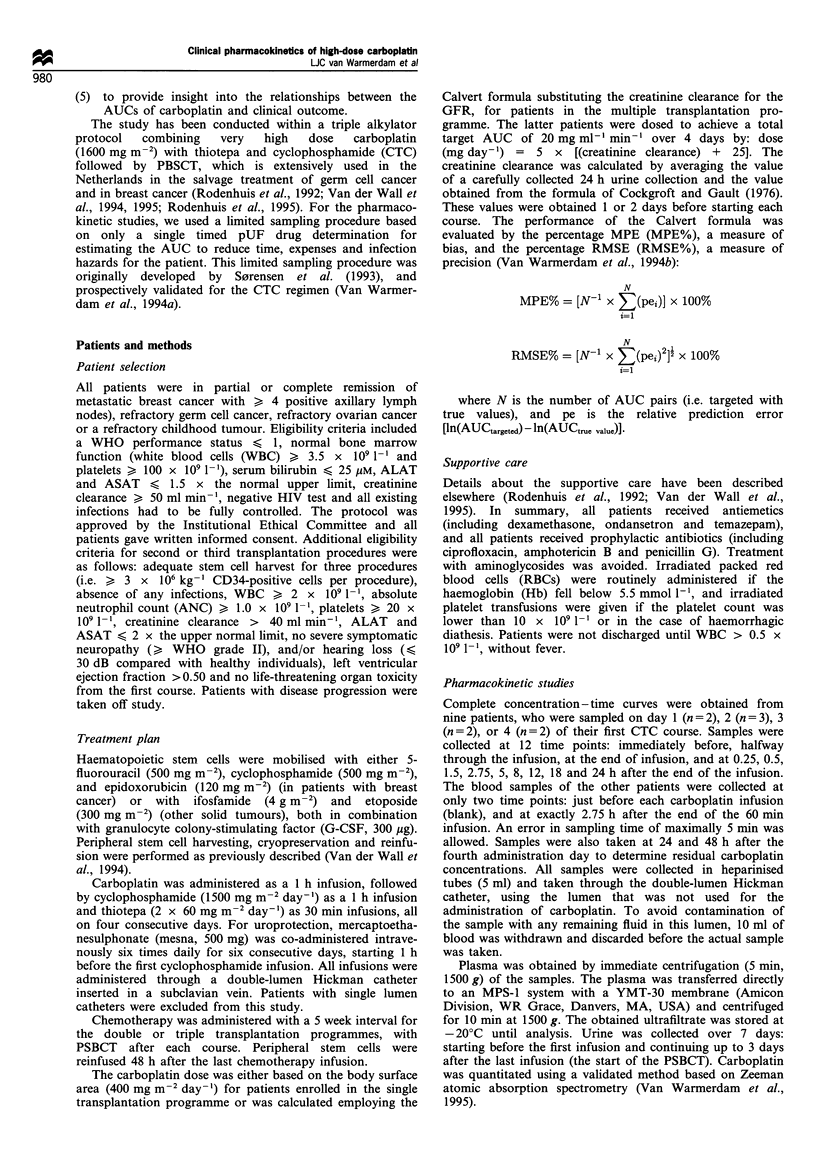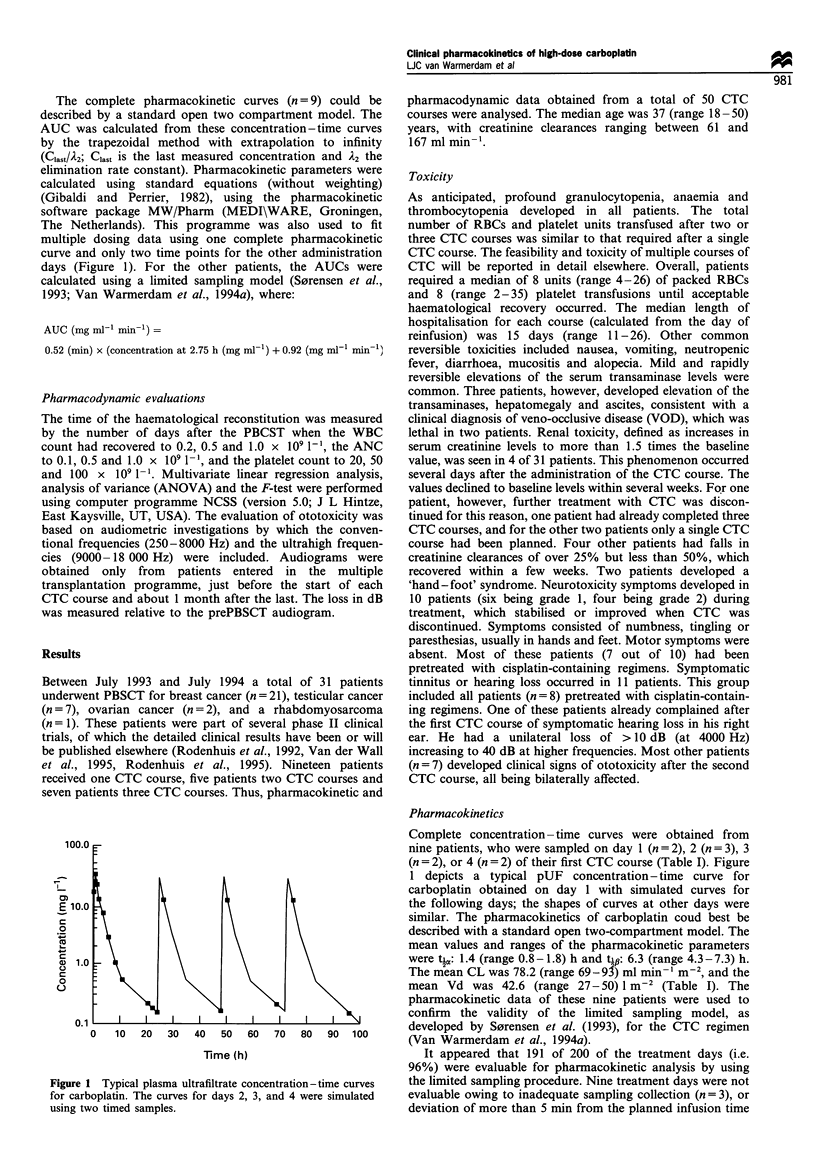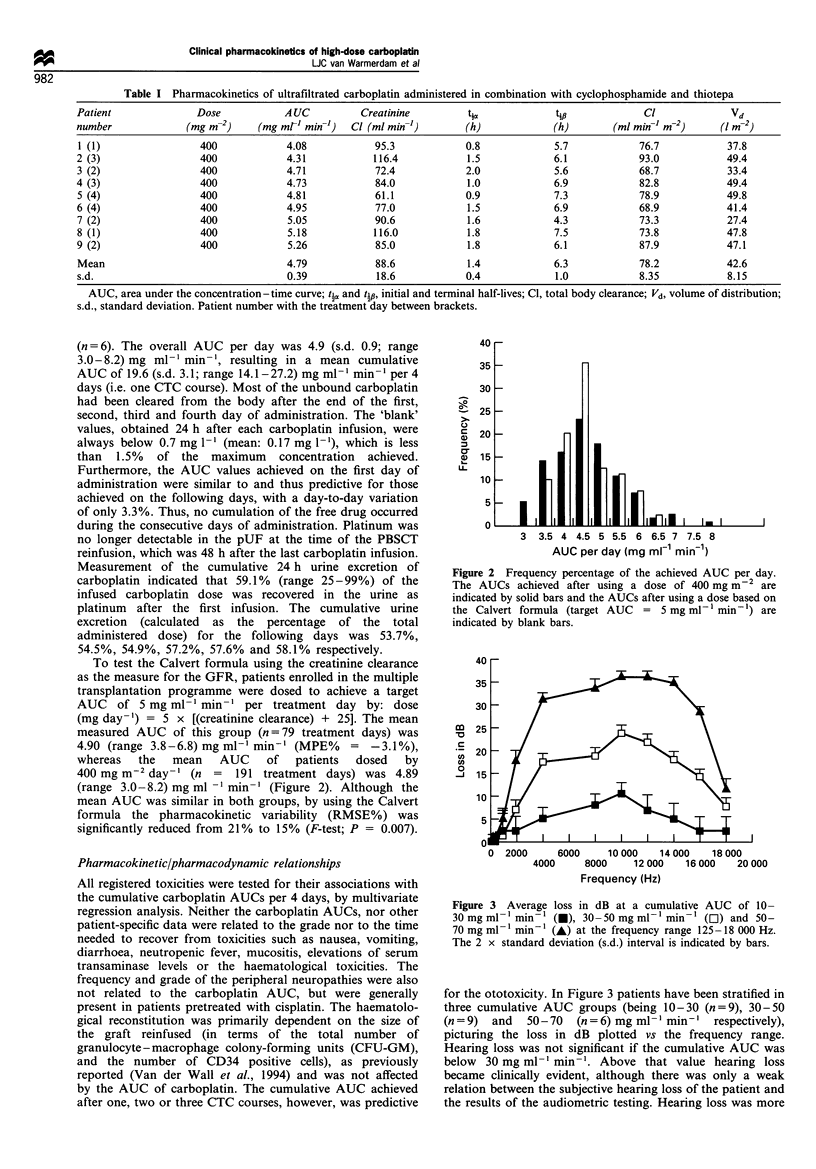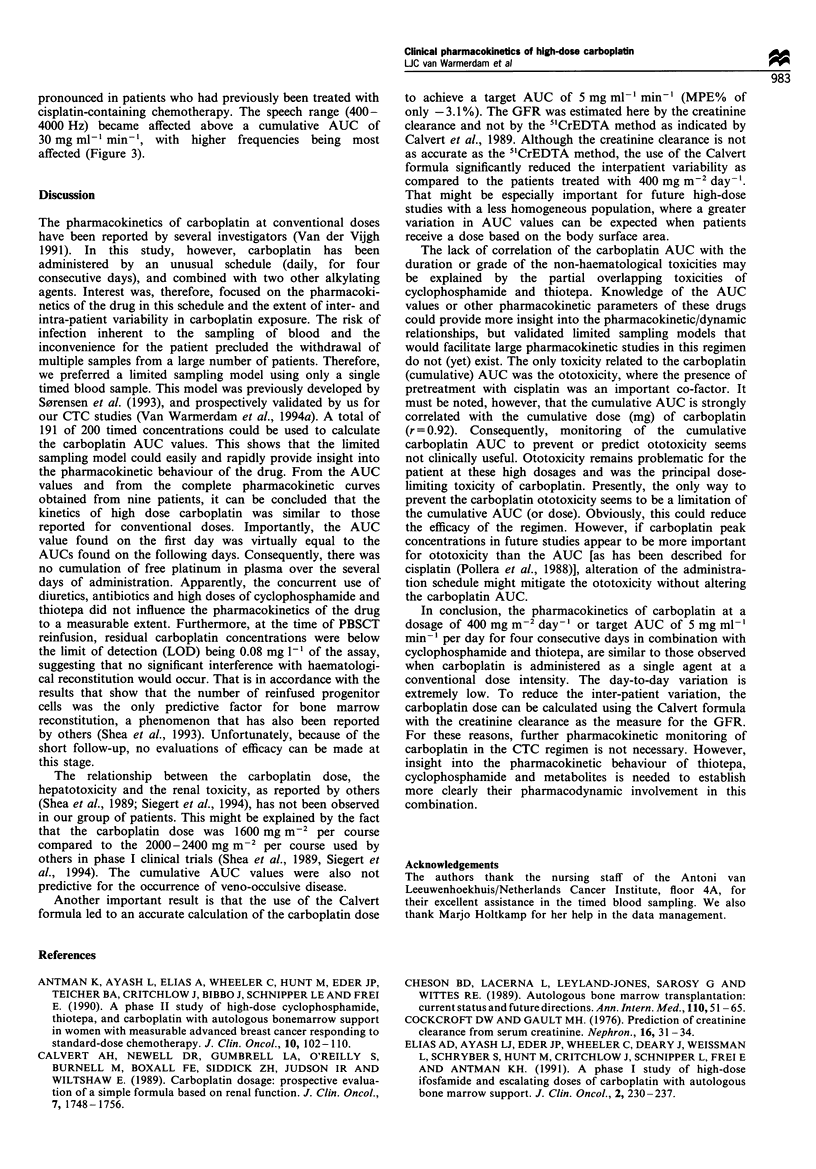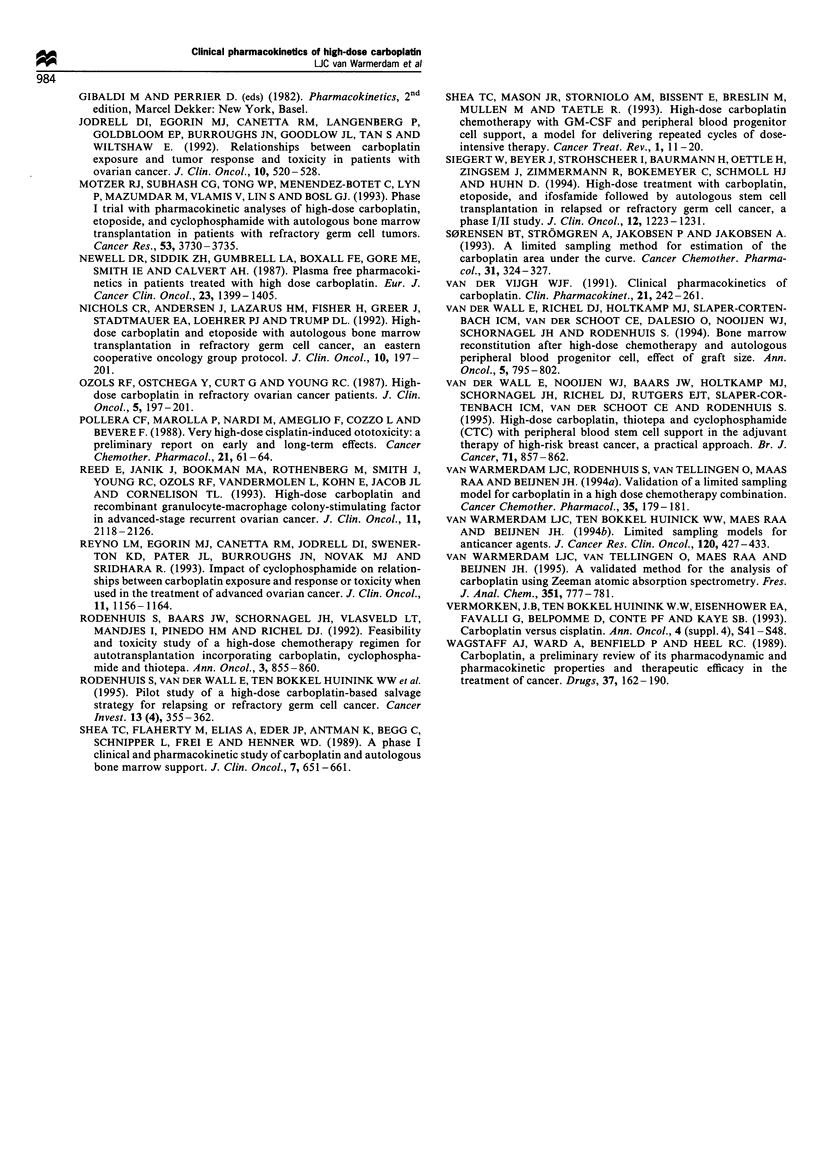# Pharmacokinetics and pharmacodynamics of carboplatin administered in a high-dose combination regimen with thiotepa, cyclophosphamide and peripheral stem cell support.

**DOI:** 10.1038/bjc.1996.191

**Published:** 1996-04

**Authors:** L. J. van Warmerdam, S. Rodenhuis, E. van der Wall, R. A. Maes, J. H. Beijnen

**Affiliations:** Department of Medical Oncology, Antoni van Leeuwenhoek Hospital, The Netherlands Cancer Institute, Amsterdam, The Netherlands.

## Abstract

The aim of this pharmacokinetic/pharmacodynamic study was to define the relationships of the carboplatin exposure with the toxicity in patients treated with high dose carboplatin (400 mg m-2 day-1), cyclophosphamide (1500 mg m-2 day-1) and thiotepa (120 mg m-2 day-1) for four consecutive days, followed by peripheral stem cell transplantation. Exposure to carboplatin was studied in 200 treatment days by measuring the area under the carboplatin plasma ultrafiltrate (pUF) concentration vs time curve (AUC). The AUC was obtained by using a previously validated limited sampling model. A total of 31 patients was studied who received one, two or three courses of this high-dose chemotherapy regimen. The unbound, plasma ultrafiltrate carboplatin was almost completely cleared from the body before each next treatment day in a course; the day-to-day AUC variation was 3.3%. The mean cumulative AUC over 4 days was 19.6 (range 14.1-27.2) mg ml-1 min-1. In 97 treatment days the carboplatin dose was calculated using the Calvert formula with the creatinine clearance as the measure for the glomerular filtration rate (GFR). For these courses, the inter-patient variability in pharmacokinetics was significantly reduced from 21% to 15% (P = 0.007) in comparison with the schemes where it was given as a fixed dose of 400 mg m-2. There were no relationships found between toxicity and the AUC of carboplatin, which may be due to the influence of overlapping toxicities of cyclophosphamide and thiotepa. However, the ototoxicity was strongly related to the cumulative carboplatin AUC. This toxicity was dose limiting for carboplatin in this schedule. It appeared that the carboplatin pharmacokinetics in these regimens were similar to those reported at conventional dosages. To reduce the inter-patient variation, the carboplatin dose can be calculated using the Calvert-formula with the creatinine clearance as the measure for the GFR.


British Journal of Cancer (1996) 73, 979-984
© 1996 Stockton Press All rights reserved 0007-0920/96 $12.00

### Pharmacokinetics and pharmacodynamics of carboplatin administered in a high-dose combination regimen with thiotepa, cyclophosphamide and peripheral stem cell support

LJC van Warmerdam[1,2], S Rodenhuis[1], E van der Wall[1], RAA Maes[3] and JH Beijnen[1,2,3]

[1]*Department of Medical Oncology, Antoni van Leeuwenhoek Hospital, The Netherlands Cancer Institute, Amsterdam, The Netherlands;* [2]*Department of Pharmacy, Slotervaart Hospital, Amsterdam, The Netherlands;* [3]*Department of Pharmaceutical Analysis and Toxicology, Faculty of Pharmacy, State University of Utrecht, Utrecht, The Netherlands.*

**Summary** The aim of this pharmacokinetic/pharmacodynamic study was to define the relationships of the carboplatin exposure with the toxicity in patients treated with high dose carboplatin (400 mg $m^{-2}$ $day^{-1}$), cyclophosphamide (1500 mg $m^{-2}$ $day^{-1}$) and thiotepa (120 mg $m^{-2}$ $day^{-1}$) for four consecutive days, followed by peripheral stem cell transplantation. Exposure to carboplatin was studied in 200 treatment days by measuring the area under the carboplatin plasma ultrafiltrate (pUF) concentration *vs* time curve (AUC). The AUC was obtained by using a previously validated limited sampling model. A total of 31 patients was studied who received one, two or three courses of this high-dose chemotherapy reigmen. The unbound, plasma ultrafiltrate carboplatin was almost completely cleared from the body before each next treatment day in a course; the day-to-day AUC variation was 3.3%. The mean cumulative AUC over 4 days was 19.6 (range 14.1-27.2) mg $ml^{-1}$ $min^{-1}$. In 97 treatment days the carboplatin dose was calculated using the Calvert formula with the creatinine clearance as the measure for the glomerular filtration rate (GFR). For these courses, the inter-patient variability in pharmacokinetics was significantly reduced from 21% to 15% ($P$ = 0.007) in comparison with the schemes where it was given as a fixed dose of 400 mg $m^{-2}$. There were no relationsips found between toxicity and the AUC of carboplatin, which may be due to the influence of overlapping toxicities of cyclophosphamide and thiotepa. However, the ototoxicity was strongly related to the cumulative carboplatin AUC. This toxicity was dose limiting for carboplatin in this schedule. It appeared that the carboplatin pharmacokinetics in these regimens were similar to those reported at conventional dosages. To reduce the inter-patient variation, the carboplatin dose can be calculated using the Calvert-formula with the creatinine clearance as the measure for the GFR.

**Keywords:** carboplatin; high-dose chemotherapy; pharmacokinetics; pharmacodynamics; peripheral stem cell transplantation

Carboplatin (*cis*-diammine 1,1-cyclobutane dicarboxylate platinum (II), CBDCA, JM8, NCS-241240, Paraplatin) is a second-generation platinum-containing chemotherapeutic compound, with established activity against a variety of solid tumours (Wagstaff *et al.*, 1989). Given at the conventional dose of 350-400 mg $m^{-2}$ every 4 weeks, carboplatin is much less nephrotoxic, neurotoxic and emetogenic than its parent compound cisplatin (Vermoken *et al.*, 1993). Its dose-limiting toxicity is myelosuppression, predominantly thrombocytopenia. It has been reported that the degree of myelosuppression is related to the exposure to carboplatin, expressed by the area under the plasma ultrafiltrate (pUF) concentration *vs* time curve (AUC) (Calvert *et al.*, 1989; Van der Vijgh 1991; Jodrell *et al.*, 1992; Reyno *et al.*, 1993). The relationship between the clearance of the drug and the glomerular filtration rate (GFR) has led to the development of a formula by which the dose can be calculated that results in a certain exposure (target AUC) to carboplatin (Calvert *et al.*, 1989). By using this formula, approximately equal AUCs can be achieved in each patient, yielding better predictive toxicity, and, possibly, higher efficacy in patients with a high GFR.

In contrast to experiences with cisplatin, it has been shown that with appropriate haematological support further escalation of the carboplatin dose is possible to 800 mg $m^{-2}$ and higher (Reed *et al.*, 1993; Ozols *et al.*, 1987; Motzer *et al.*, 1993; Newell *et al.*, 1987; Shea *et al.*, 1989, 1993; Nichols *et al.*, 1992). The use of higher doses of chemotherapy may be beneficial, since it has been shown to provide more long-term remissions in poor risk patients with various malignancies (Ozols *et al.*, 1987; Shea *et al.*, 1989; Nichols *et al.*, 1992; Siegert *et al.*, 1994; Antman *et al.*, 1990; Cheson *et al.*, 1989). The relative lack of non-haematological toxicities makes carboplatin an attractive drug in the setting of high-dose chemotherapy, where recovery from myelosuppression can be accomplished by autologous bone marrow transplantation or peripheral blood stem cell transplantation (PBSCT). However, when very high doses carboplatin are administered, non-haematological toxicities such as neurotoxicity, ototoxicity and nephrotoxicity become dose limiting (Ozols *et al.*, 1987; Nichols *et al.*, 1992; Siegert *et al.*, 1994; Elias *et al.*, 1991). The degree of these toxicities is difficult to predict and their extents vary considerably among patients (Ozols *et al.*, 1987; Motzer *et al.*, 1993; Newell *et al.*, 1987; Nichols *et al.*, 1992). A plausible explanation might be the interindividual difference in exposure to the drug (AUC), as has been established at conventional doses for the myelosuppression. However, at very high doses, information about the pharmacokinetics of carboplatin and the relationships between the AUC and the dose-limiting toxicities are scarce. Therefore, we initiated a pharmacokinetic/pharmacodynamic study, with the following aims:

(1) to determine the pharmacokinetic behaviour of carboplatin administered over four consecutive days;
(2) to test the day-to-day variation in drug exposure (AUC), and to measure the possible occurrence of drug accumulation;
(3) to determine the residual free carboplatin fraction before the reinfusion of stem cells;
(4) to test the Calvert formula in this setting using the creatinine clearance as a measure for the GFR;

Correspondence: LJC van Warmerdam, Department of Pharmacy, Slotervaart Hospital, Louwesweg 6, 1066 EC, Amsterdam, The Netherlands
Received 5 June 1995; revised 28 September 1995; accepted 15 November 1995

(5) to provide insight into the relationships between the AUCs of carboplatin and clinical outcome.

The study has been conducted within a triple alkylator protocol combining very high dose carboplatin (1600 mg $m^{-2}$) with thiotepa and cyclophosphamide (CTC) followed by PBSCT, which is extensively used in the Netherlands in the salvage treatment of germ cell cancer and in breast cancer (Rodenhuis *et al.*, 1992; Van der Wall *et al.*, 1994, 1995; Rodenhuis *et al.*, 1995). For the pharmacokinetic studies, we used a limited sampling procedure based on only a single timed pUF drug determination for estimating the AUC to reduce time, expenses and infection hazards for the patient. This limited sampling procedure was originally developed by Sørensen *et al.* (1993), and prospectively validated for the CTC regimen (Van Warmerdam *et al.*, 1994*a*).

### Patients and methods

### *Patient selection*

All patients were in partial or complete remission of metastatic breast cancer with $\geqslant$ 4 positive axillary lymph nodes), refractory germ cell cancer, refractory ovarian cancer or a refractory childhood tumour. Eligibility criteria included a WHO performance status $\leqslant$ 1, normal bone marrow function (white blood cells (WBC) $\geqslant 3.5 \times 10^9$ $l^{-1}$ and platelets $\geqslant 100 \times 10^9$ $l^{-1}$), serum bilirubin $\leqslant$ 25 $\mu$M, ALAT and ASAT $\leqslant$ 1.5 $\times$ the normal upper limit, creatinine clearance $\geqslant$ 50 ml $min^{-1}$, negative HIV test and all existing infections had to be fully controlled. The protocol was approved by the Institutional Ethical Committee and all patients gave written informed consent. Additional eligibility criteria for second or third transplantation procedures were as follows: adequate stem cell harvest for three procedures (i.e. $\geqslant 3 \times 10^6$ $kg^{-1}$ CD34-positive cells per procedure), absence of any infections, WBC $\geqslant 2 \times 10^9$ $l^{-1}$, absolute neutrophil count (ANC) $\geqslant 1.0 \times 10^9$ $l^{-1}$, platelets $\geqslant 20 \times 10^9$ $l^{-1}$, creatinine clearance > 40 ml $min^{-1}$, ALAT and ASAT $\leqslant$ 2 $\times$ the upper normal limit, no severe symptomatic neuropathy ($\geqslant$ WHO grade II), and/or hearing loss ($\leqslant$ 30 dB compared with healthy individuals), left ventricular ejection fraction >0.50 and no life-threatening organ toxicity from the first course. Patients with disease progression were taken off study.

### *Treatment plan*

Haematopoietic stem cells were mobilised with either 5-fluorouracil (500 mg $m^{-2}$), cyclophosphamide (500 mg $m^{-2}$), and epidoxorubicin (120 mg $m^{-2}$) (in patients with breast cancer) or with ifosfamide (4 g $m^{-2}$) and etoposide (300 mg $m^{-2}$) (other solid tumours), both in combination with granulocyte colony-stimulating factor (G-CSF, 300 $\mu$g). Peripheral stem cell harvesting, cryopreservation and reinfusion were performed as previously described (Van der Wall *et al.*, 1994).

Carboplatin was administered as a 1 h infusion, followed by cyclophosphamide (1500 mg $m^{-2}$ $day^{-1}$) as a 1 h infusion and thiotepa (2 $\times$ 60 mg $m^{-2}$ $day^{-1}$) as 30 min infusions, all on four consecutive days. For uroprotection, mercaptoethanesulphonate (mesna, 500 mg) was co-administered intravenously six times daily for six consecutive days, starting 1 h before the first cyclophosphamide infusion. All infusions were administered through a double-lumen Hickman catheter inserted in a subclavian vein. Patients with single lumen catheters were excluded from this study.

Chemotherapy was administered with a 5 week interval for the double or triple transplantation programmes, with PSBCT after each course. Peripheral stem cells were reinfused 48 h after the last chemotherapy infusion.

The carboplatin dose was either based on the body surface area (400 mg $m^{-2}$ $day^{-1}$) for patients enrolled in the single transplantation programme or was calculated employing the Calvert formula substituting the creatinine clearance for the GFR, for patients in the multiple transplantation programme. The latter patients were dosed to achieve a total target AUC of 20 mg $ml^{-1}$ $min^{-1}$ over 4 days by: dose (mg $day^{-1}$) = 5 $\times$ [(creatinine clearance) + 25]. The creatinine clearance was calculated by averaging the value of a carefully collected 24 h urine collection and the value obtained from the formula of Cockgroft and Gault (1976). These values were obtained 1 or 2 days before starting each course. The performance of the Calvert formula was evaluated by the percentage MPE (MPE%), a measure of bias, and the percentage RMSE (RMSE%), a measure of precision (Van Warmerdam *et al.*, 1994*b*):

$$\text{MPE\%} = [N^{-1} \times \sum_{i=1}^{N}(\text{pe}_i)] \times 100\%$$

$$\text{RMSE\%} = [N^{-1} \times \sum_{i=1}^{N}(\text{pe}_i)^2]^{\frac{1}{2}} \times 100\%$$

where $N$ is the number of AUC pairs (i.e. targeted with true values), and pe is the relative prediction error $[\ln(\text{AUC}_{\text{targeted}}) - \ln(\text{AUC}_{\text{true value}})]$.

### *Supportive care*

Details about the supportive care have been described elsewhere (Rodenhuis *et al.*, 1992; Van der Wall *et al.*, 1995). In summary, all patients received antiemetics (including dexamethasone, ondansetron and temazepam), and all patients received prophylactic antibiotics (including ciprofloxacin, amphotericin B and penicillin G). Treatment with aminoglycosides was avoided. Irradiated packed red blood cells (RBCs) were routinely administered if the haemoglobin (Hb) fell below 5.5 mmol $l^{-1}$, and irradiated platelet transfusions were given if the platelet count was lower than $10 \times 10^9$ $l^{-1}$ or in the case of haemorrhagic diathesis. Patients were not discharged until WBC > 0.5 $\times$ $10^9$ $l^{-1}$, without fever.

### *Pharmacokinetic studies*

Complete concentration–time curves were obtained from nine patients, who were sampled on day 1 ($n$=2), 2 ($n$=3), 3 ($n$=2), or 4 ($n$=2) of their first CTC course. Samples were collected at 12 time points: immediately before, halfway through the infusion, at the end of infusion, and at 0.25, 0.5, 1.5, 2.75, 5, 8, 12, 18 and 24 h after the end of the infusion. The blood samples of the other patients were collected at only two time points: just before each carboplatin infusion (blank), and at exactly 2.75 h after the end of the 60 min infusion. An error in sampling time of maximally 5 min was allowed. Samples were also taken at 24 and 48 h after the fourth administration day to determine residual carboplatin concentrations. All samples were collected in heparinised tubes (5 ml) and taken through the double-lumen Hickman catheter, using the lumen that was not used for the administration of carboplatin. To avoid contamination of the sample with any remaining fluid in this lumen, 10 ml of blood was withdrawn and discarded before the actual sample was taken.

Plasma was obtained by immediate centrifugation (5 min, 1500 ***g***) of the samples. The plasma was transferred directly to an MPS-1 system with a YMT-30 membrane (Amicon Division, WR Grace, Danvers, MA, USA) and centrifuged for 10 min at 1500 ***g***. The obtained ultrafiltrate was stored at −20°C until analysis. Urine was collected over 7 days: starting before the first infusion and continuing up to 3 days after the last infusion (the start of the PSBCT). Carboplatin was quantitated using a validated method based on Zeeman atomic absorption spectrometry (Van Warmerdam *et al.*, 1995).

The complete pharmacokinetic curves ($n = 9$) could be described by a standard open two compartment model. The AUC was calculated from these concentration–time curves by the trapezoidal method with extrapolation to infinity ($C_{last}/\lambda_2$; $C_{last}$ is the last measured concentration and $\lambda_2$ the elimination rate constant). Pharmacokinetic parameters were calculated using standard equations (without weighting) (Gibaldi and Perrier, 1982), using the pharmacokinetic software package MW/Pharm (MEDI\WARE, Groningen, The Netherlands). This programme was also used to fit multiple dosing data using one complete pharmacokinetic curve and only two time points for the other administration days (Figure 1). For the other patients, the AUCs were calculated using a limited sampling model (Sørensen *et al.*, 1993; Van Warmerdam *et al.*, 1994*a*), where:

$$\text{AUC (mg ml}^{-1}\text{ min}^{-1}) =$$

$$0.52\ (\text{min}) \times (\text{concentration at 2.75 h (mg ml}^{-1})) + 0.92\ (\text{mg ml}^{-1}\text{ min}^{-1})$$

*Pharmacodynamic evaluations*

The time of the haematological reconstitution was measured by the number of days after the PBCST when the WBC count had recovered to 0.2, 0.5 and 1.0 × $10^9$ $l^{-1}$, the ANC to 0.1, 0.5 and 1.0 × $10^9$ $l^{-1}$, and the platelet count to 20, 50 and 100 × $10^9$ $l^{-1}$. Multivariate linear regression analysis, analysis of variance (ANOVA) and the *F*-test were performed using computer programme NCSS (version 5.0; J L Hintze, East Kaysville, UT, USA). The evaluation of ototoxicity was based on audiometric investigations by which the conventional frequencies (250–8000 Hz) and the ultrahigh frequencies (9000–18 000 Hz) were included. Audiograms were obtained only from patients entered in the multiple transplantation programme, just before the start of each CTC course and about 1 month after the last. The loss in dB was measured relative to the prePBSCT audiogram.

### Results

Between July 1993 and July 1994 a total of 31 patients underwent PBSCT for breast cancer ($n = 21$), testicular cancer ($n = 7$), ovarian cancer ($n = 2$), and a rhabdomyosarcoma ($n = 1$). These patients were part of several phase II clinical trials, of which the detailed clinical results have been or will be published elsewhere (Rodenhuis *et al.*, 1992, Van der Wall *et al.*, 1995, Rodenhuis *et al.*, 1995). Nineteen patients received one CTC course, five patients two CTC courses and seven patients three CTC courses. Thus, pharmacokinetic and pharmacodynamic data obtained from a total of 50 CTC courses were analysed. The median age was 37 (range 18–50) years, with creatinine clearances ranging between 61 and 167 ml $min^{-1}$.

**Figure 1** Typical plasma ultrafiltrate concentration–time curves for carboplatin. The curves for days 2, 3, and 4 were simulated using two timed samples.

*Toxicity*

As anticipated, profound granulocytopenia, anaemia and thrombocytopenia developed in all patients. The total number of RBCs and platelet units transfused after two or three CTC courses was similar to that required after a single CTC course. The feasibility and toxicity of multiple courses of CTC will be reported in detail elsewhere. Overall, patients required a median of 8 units (range 4–26) of packed RBCs and 8 (range 2–35) platelet transfusions until acceptable haematological recovery occurred. The median length of hospitalisation for each course (calculated from the day of reinfusion) was 15 days (range 11–26). Other common reversible toxicities included nausea, vomiting, neutropenic fever, diarrhoea, mucositis and alopecia. Mild and rapidly reversible elevations of the serum transaminase levels were common. Three patients, however, developed elevation of the transaminases, hepatomegaly and ascites, consistent with a clinical diagnosis of veno-occlusive disease (VOD), which was lethal in two patients. Renal toxicity, defined as increases in serum creatinine levels to more than 1.5 times the baseline value, was seen in 4 of 31 patients. This phenomenon occurred several days after the administration of the CTC course. The values declined to baseline levels within several weeks. For one patient, however, further treatment with CTC was discontinued for this reason, one patient had already completed three CTC courses, and for the other two patients only a single CTC course had been planned. Four other patients had falls in creatinine clearances of over 25% but less than 50%, which recovered within a few weeks. Two patients developed a 'hand–foot' syndrome. Neurotoxicity symptoms developed in 10 patients (six being grade 1, four being grade 2) during treatment, which stabilised or improved when CTC was discontinued. Symptoms consisted of numbness, tingling or paresthesias, usually in hands and feet. Motor symptoms were absent. Most of these patients (7 out of 10) had been pretreated with cisplatin-containing regimens. Symptomatic tinnitus or hearing loss occurred in 11 patients. This group included all patients ($n = 8$) pretreated with cisplatin-containing regimens. One of these patients already complained after the first CTC course of symptomatic hearing loss in his right ear. He had a unilateral loss of $> 10$ dB (at 4000 Hz) increasing to 40 dB at higher frequencies. Most other patients ($n = 7$) developed clinical signs of ototoxicity after the second CTC course, all being bilaterally affected.

*Pharmacokinetics*

Complete concentration–time curves were obtained from nine patients, who were sampled on day 1 ($n = 2$), 2 ($n = 3$), 3 ($n = 2$), or 4 ($n = 2$) of their first CTC course (Table I). Figure 1 depicts a typical pUF concentration–time curve for carboplatin obtained on day 1 with simulated curves for the following days; the shapes of curves at other days were similar. The pharmacokinetics of carboplatin coud best be described with a standard open two-compartment model. The mean values and ranges of the pharmacokinetic parameters were $t_{\frac{1}{2}\alpha}$: 1.4 (range 0.8–1.8) h and $t_{\frac{1}{2}\beta}$: 6.3 (range 4.3–7.3) h. The mean CL was 78.2 (range 69–93) ml $min^{-1}$ $m^{-2}$, and the mean Vd was 42.6 (range 27–50) l $m^{-2}$ (Table I). The pharmacokinetic data of these nine patients were used to confirm the validity of the limited sampling model, as developed by Sørensen *et al.* (1993), for the CTC regimen (Van Warmerdam *et al.*, 1994*a*).

It appeared that 191 of 200 of the treatment days (i.e. 96%) were evaluable for pharmacokinetic analysis by using the limited sampling procedure. Nine treatment days were not evaluable owing to inadequate sampling collection ($n = 3$), or deviation of more than 5 min from the planned infusion time

**Table I** Pharmacokinetics of ultrafiltrated carboplatin administered in combination with cyclophosphamide and thiotepa

| Patient number | Dose ($mg\ m^{-2}$) | AUC ($mg\ ml^{-1}\ min^{-1}$) | Creatinine Cl ($ml\ min^{-1}$) | $t_{\frac{1}{2}\alpha}$ (h) | $t_{\frac{1}{2}\beta}$ (h) | Cl ($ml\ min^{-1}\ m^{-2}$) | $V_d$ ($l\ m^{-2}$) |
|---|---|---|---|---|---|---|---|
| 1 (1) | 400 | 4.08 | 95.3 | 0.8 | 5.7 | 76.7 | 37.8 |
| 2 (3) | 400 | 4.31 | 116.4 | 1.5 | 6.1 | 93.0 | 49.4 |
| 3 (2) | 400 | 4.71 | 72.4 | 2.0 | 5.6 | 68.7 | 33.4 |
| 4 (3) | 400 | 4.73 | 84.0 | 1.0 | 6.9 | 82.8 | 49.4 |
| 5 (4) | 400 | 4.81 | 61.1 | 0.9 | 7.3 | 78.9 | 49.8 |
| 6 (4) | 400 | 4.95 | 77.0 | 1.5 | 6.9 | 68.9 | 41.4 |
| 7 (2) | 400 | 5.05 | 90.6 | 1.6 | 4.3 | 73.3 | 27.4 |
| 8 (1) | 400 | 5.18 | 116.0 | 1.8 | 7.5 | 73.8 | 47.8 |
| 9 (2) | 400 | 5.26 | 85.0 | 1.8 | 6.1 | 87.9 | 47.1 |
| Mean | | 4.79 | 88.6 | 1.4 | 6.3 | 78.2 | 42.6 |
| s.d. | | 0.39 | 18.6 | 0.4 | 1.0 | 8.35 | 8.15 |

AUC, area under the concentration–time curve; $t_{\frac{1}{2}\alpha}$ and $t_{\frac{1}{2}\beta}$, initial and terminal half-lives; Cl, total body clearance; $V_d$, volume of distribution; s.d., standard deviation. Patient number with the treatment day between brackets.

($n = 6$). The overall AUC per day was 4.9 (s.d. 0.9; range 3.0–8.2) mg $ml^{-1}$ $min^{-1}$, resulting in a mean cumulative AUC of 19.6 (s.d. 3.1; range 14.1–27.2) mg $ml^{-1}$ $min^{-1}$ per 4 days (i.e. one CTC course). Most of the unbound carboplatin had been cleared from the body after the end of the first, second, third and fourth day of administration. The 'blank' values, obtained 24 h after each carboplatin infusion, were always below 0.7 mg $l^{-1}$ (mean: 0.17 mg $l^{-1}$), which is less than 1.5% of the maximum concentration achieved. Furthermore, the AUC values achieved on the first day of administration were similar to and thus predictive for those achieved on the following days, with a day-to-day variation of only 3.3%. Thus, no cumulation of the free drug occurred during the consecutive days of administration. Platinum was no longer detectable in the pUF at the time of the PBSCT reinfusion, which was 48 h after the last carboplatin infusion. Measurement of the cumulative 24 h urine excretion of carboplatin indicated that 59.1% (range 25–99%) of the infused carboplatin dose was recovered in the urine as platinum after the first infusion. The cumulative urine excretion (calculated as the percentage of the total administered dose) for the following days was 53.7%, 54.5%, 54.9%, 57.2%, 57.6% and 58.1% respectively.

To test the Calvert formula using the creatinine clearance as the measure for the GFR, patients enrolled in the multiple transplantation programme were dosed to achieve a target AUC of 5 mg $ml^{-1}$ $min^{-1}$ per treatment day by: dose (mg $day^{-1}$) = 5 × [(creatinine clearance) + 25]. The mean measured AUC of this group ($n = 79$ treatment days) was 4.90 (range 3.8–6.8) mg $ml^{-1}$ $min^{-1}$ (MPE% = −3.1%), whereas the mean AUC of patients dosed by 400 mg $m^{-2}$ $day^{-1}$ ($n$ = 191 treatment days) was 4.89 (range 3.0–8.2) mg ml $^{-1}$ $min^{-1}$ (Figure 2). Although the mean AUC was similar in both groups, by using the Calvert formula the pharmacokinetic variability (RMSE%) was significantly reduced from 21% to 15% ($F$-test; $P$ = 0.007).

*Pharmacokinetic/pharmacodynamic relationships*

All registered toxicities were tested for their associations with the cumulative carboplatin AUCs per 4 days, by multivariate regression analysis. Neither the carboplatin AUCs, nor other patient-specific data were related to the grade nor to the time needed to recover from toxicities such as nausea, vomiting, diarrhoea, neutropenic fever, mucositis, elevations of serum transaminase levels or the haematological toxicities. The frequency and grade of the peripheral neuropathies were also not related to the carboplatin AUC, but were generally present in patients pretreated with cisplatin. The haematological reconstitution was primarily dependent on the size of the graft reinfused (in terms of the total number of granulocyte–macrophage colony-forming units (CFU-GM), and the number of CD34 positive cells), as previously reported (Van der Wall et al., 1994) and was not affected by the AUC of carboplatin. The cumulative AUC achieved after one, two or three CTC courses, however, was predictive for the ototoxicity. In Figure 3 patients have been stratified in three cumulative AUC groups (being 10–30 ($n = 9$), 30–50 ($n = 9$) and 50–70 ($n = 6$) mg $ml^{-1}$ $min^{-1}$ respectively), picturing the loss in dB plotted *vs* the frequency range. Hearing loss was not significant if the cumulative AUC was below 30 mg $ml^{-1}$ $min^{-1}$. Above that value hearing loss became clinically evident, although there was only a weak relation between the subjective hearing loss of the patient and the results of the audiometric testing. Hearing loss was more

**Figure 2** Frequency percentage of the achieved AUC per day. The AUCs achieved after using a dose of 400 mg $m^{-2}$ are indicated by solid bars and the AUCs after using a dose based on the Calvert formula (target AUC = 5 mg $ml^{-1}$ $min^{-1}$) are indicated by blank bars.

**Figure 3** Average loss in dB at a cumulative AUC of 10–30 mg $ml^{-1}$ $min^{-1}$ (■), 30–50 mg $ml^{-1}$ $min^{-1}$ (□) and 50–70 mg $ml^{-1}$ $min^{-1}$ (▲) at the frequency range 125–18 000 Hz. The 2 × standard deviation (s.d.) interval is indicated by bars.

pronounced in patients who had previously been treated with cisplatin-containing chemotherapy. The speech range (400–4000 Hz) became affected above a cumulative AUC of 30 mg $ml^{-1}$ $min^{-1}$, with higher frequencies being most affected (Figure 3).

### Discussion

The pharmacokinetics of carboplatin at conventional doses have been reported by several investigators (Van der Vijgh 1991). In this study, however, carboplatin has been administered by an unusual schedule (daily, for four consecutive days), and combined with two other alkylating agents. Interest was, therefore, focused on the pharmacokinetics of the drug in this schedule and the extent of inter- and intra-patient variability in carboplatin exposure. The risk of infection inherent to the sampling of blood and the inconvenience for the patient precluded the withdrawal of multiple samples from a large number of patients. Therefore, we preferred a limited sampling model using only a single timed blood sample. This model was previously developed by Sørensen *et al.* (1993), and prospectively validated by us for our CTC studies (Van Warmerdam *et al.*, 1994*a*). A total of 191 of 200 timed concentrations could be used to calculate the carboplatin AUC values. This shows that the limited sampling model could easily and rapidly provide insight into the pharmacokinetic behaviour of the drug. From the AUC values and from the complete pharmacokinetic curves obtained from nine patients, it can be concluded that the kinetics of high dose carboplatin was similar to those reported for conventional doses. Importantly, the AUC value found on the first day was virtually equal to the AUCs found on the following days. Consequently, there was no cumulation of free platinum in plasma over the several days of administration. Apparently, the concurrent use of diuretics, antibiotics and high doses of cyclophosphamide and thiotepa did not influence the pharmacokinetics of the drug to a measurable extent. Furthermore, at the time of PBSCT reinfusion, residual carboplatin concentrations were below the limit of detection (LOD) being 0.08 mg $l^{-1}$ of the assay, suggesting that no significant interference with haematological reconstitution would occur. That is in accordance with the results that show that the number of reinfused progenitor cells was the only predictive factor for bone marrow reconstitution, a phenomenon that has also been reported by others (Shea *et al.*, 1993). Unfortunately, because of the short follow-up, no evaluations of efficacy can be made at this stage.

The relationship between the carboplatin dose, the hepatotoxicity and the renal toxicity, as reported by others (Shea *et al.*, 1989; Siegert *et al.*, 1994), has not been observed in our group of patients. This might be explained by the fact that the carboplatin dose was 1600 mg $m^{-2}$ per course compared to the 2000–2400 mg $m^{-2}$ per course used by others in phase I clinical trials (Shea *et al.*, 1989, Siegert *et al.*, 1994). The cumulative AUC values were also not predictive for the occurrence of veno-occulsive disease.

Another important result is that the use of the Calvert formula led to an accurate calculation of the carboplatin dose to achieve a target AUC of 5 mg $ml^{-1}$ $min^{-1}$ (MPE% of only −3.1%). The GFR was estimated here by the creatinine clearance and not by the $^{51}$CrEDTA method as indicated by Calvert *et al.*, 1989. Although the creatinine clearance is not as accurate as the $^{51}$CrEDTA method, the use of the Calvert formula significantly reduced the interpatient variability as compared to the patients treated with 400 mg $m^{-2}$ $day^{-1}$. That might be especially important for future high-dose studies with a less homogeneous population, where a greater variation in AUC values can be expected when patients receive a dose based on the body surface area.

The lack of correlation of the carboplatin AUC with the duration or grade of the non-haematological toxicities may be explained by the partial overlapping toxicities of cyclophosphamide and thiotepa. Knowledge of the AUC values or other pharmacokinetic parameters of these drugs could provide more insight into the pharmacokinetic/dynamic relationships, but validated limited sampling models that would facilitate large pharmacokinetic studies in this regimen do not (yet) exist. The only toxicity related to the carboplatin (cumulative) AUC was the ototoxicity, where the presence of pretreatment with cisplatin was an important co-factor. It must be noted, however, that the cumulative AUC is strongly correlated with the cumulative dose (mg) of carboplatin ($r=0.92$). Consequently, monitoring of the cumulative carboplatin AUC to prevent or predict ototoxicity seems not clinically useful. Ototoxicity remains problematic for the patient at these high dosages and was the principal dose-limiting toxicity of carboplatin. Presently, the only way to prevent the carboplatin ototoxicity seems to be a limitation of the cumulative AUC (or dose). Obviously, this could reduce the efficacy of the regimen. However, if carboplatin peak concentrations in future studies appear to be more important for ototoxicity than the AUC [as has been described for cisplatin (Pollera *et al.*, 1988)], alteration of the administration schedule might mitigate the ototoxicity without altering the carboplatin AUC.

In conclusion, the pharmacokinetics of carboplatin at a dosage of 400 mg $m^{-2}$ $day^{-1}$ or target AUC of 5 mg $ml^{-1}$ $min^{-1}$ per day for four consecutive days in combination with cyclophosphamide and thiotepa, are similar to those observed when carboplatin is administered as a single agent at a conventional dose intensity. The day-to-day variation is extremely low. To reduce the inter-patient variation, the carboplatin dose can be calculated using the Calvert formula with the creatinine clearance as the measure for the GFR. For these reasons, further pharmacokinetic monitoring of carboplatin in the CTC regimen is not necessary. However, insight into the pharmacokinetic behaviour of thiotepa, cyclophosphamide and metabolites is needed to establish more clearly their pharmacodynamic involvement in this combination.

**Acknowledgements**
The authors thank the nursing staff of the Antoni van Leeuwenhoekhuis/Netherlands Cancer Institute, floor 4A, for their excellent assistance in the timed blood sampling. We also thank Marjo Holtkamp for her help in the data management.

### References

ANTMAN K, AYASH L, ELIAS A, WHEELER C, HUNT M, EDER JP, TEICHER BA, CRITCHLOW J, BIBBO J, SCHNIPPER LE AND FREI E. (1990). A phase II study of high-dose cyclophosphamide, thiotepa, and carboplatin with autologous bonemarrow support in women with measurable advanced breast cancer responding to standard-dose chemotherapy. *J. Clin. Oncol.*, **10**, 102–110.

CALVERT AH, NEWELL DR, GUMBRELL LA, O'REILLY S, BURNELL M, BOXALL FE, SIDDICK ZH, JUDSON IR AND WILTSHAW E. (1989). Carboplatin dosage: prospective evaluation of a simple formula based on renal function. *J. Clin. Oncol.*, **7**, 1748–1756.

CHESON BD, LACERNA L, LEYLAND-JONES, SAROSY G AND WITTES RE. (1989). Autologous bone marrow transplantation: current status and future directions. *Ann. Intern. Med.*, **110**, 51–65.

COCKCROFT DW AND GAULT MH. (1976). Prediction of creatinine clearance from serum creatinine. *Nephron.*, **16**, 31–34.

ELIAS AD, AYASH LJ, EDER JP, WHEELER C, DEARY J, WEISSMAN L, SCHRYBER S, HUNT M, CRITCHLOW J, SCHNIPPER L, FREI E AND ANTMAN KH. (1991). A phase I study of high-dose ifosfamide and escalating doses of carboplatin with autologous bone marrow support. *J. Clin. Oncol.*, **2**, 230–237.

GIBALDI M AND PERRIER D. (eds) (1982). *Pharmacokinetics*, 2nd edition, Marcel Dekker: New York, Basel.

JODRELL DI, EGORIN MJ, CANETTA RM, LANGENBERG P, GOLDBLOOM EP, BURROUGHS JN, GOODLOW JL, TAN S AND WILTSHAW E. (1992). Relationships between carboplatin exposure and tumor response and toxicity in patients with ovarian cancer. *J. Clin. Oncol.*, **10**, 520–528.

MOTZER RJ, SUBHASH CG, TONG WP, MENENDEZ-BOTET C, LYN P, MAZUMDAR M, VLAMIS V, LIN S AND BOSL GJ. (1993). Phase I trial with pharmacokinetic analyses of high-dose carboplatin, etoposide, and cyclophosphamide with autologous bone marrow transplantation in patients with refractory germ cell tumors. *Cancer Res.*, **53**, 3730–3735.

NEWELL DR, SIDDIK ZH, GUMBRELL LA, BOXALL FE, GORE ME, SMITH IE AND CALVERT AH. (1987). Plasma free pharmacokinetics in patients treated with high dose carboplatin. *Eur. J. Cancer Clin. Oncol.*, **23**, 1399–1405.

NICHOLS CR, ANDERSEN J, LAZARUS HM, FISHER H, GREER J, STADTMAUER EA, LOEHRER PJ AND TRUMP DL. (1992). High-dose carboplatin and etoposide with autologous bone marrow transplantation in refractory germ cell cancer, an eastern cooperative oncology group protocol. *J. Clin. Oncol.*, **10**, 197–201.

OZOLS RF, OSTCHEGA Y, CURT G AND YOUNG RC. (1987). High-dose carboplatin in refractory ovarian cancer patients. *J. Clin. Oncol.*, **5**, 197–201.

POLLERA CF, MAROLLA P, NARDI M, AMEGLIO F, COZZO L AND BEVERE F. (1988). Very high-dose cisplatin-induced ototoxicity: a preliminary report on early and long-term effects. *Cancer Chemother. Pharmacol.*, **21**, 61–64.

REED E, JANIK J, BOOKMAN MA, ROTHENBERG M, SMITH J, YOUNG RC, OZOLS RF, VANDERMOLEN L, KOHN E, JACOB JL AND CORNELISON TL. (1993). High-dose carboplatin and recombinant granulocyte-macrophage colony-stimulating factor in advanced-stage recurrent ovarian cancer. *J. Clin. Oncol.*, **11**, 2118–2126.

REYNO LM, EGORIN MJ, CANETTA RM, JODRELL DI, SWENERTON KD, PATER JL, BURROUGHS JN, NOVAK MJ AND SRIDHARA R. (1993). Impact of cyclophosphamide on relationships between carboplatin exposure and response or toxicity when used in the treatment of advanced ovarian cancer. *J. Clin. Oncol.*, **11**, 1156–1164.

RODENHUIS S, BAARS JW, SCHORNAGEL JH, VLASVELD LT, MANDJES I, PINEDO HM AND RICHEL DJ. (1992). Feasibility and toxicity study of a high-dose chemotherapy regimen for autotransplantation incorporating carboplatin, cyclophosphamide and thiotepa. *Ann. Oncol.*, **3**, 855–860.

RODENHUIS S, VAN DER WALL E, TEN BOKKEL HUININK WW *et al.* (1995). Pilot study of a high-dose carboplatin-based salvage strategy for relapsing or refractory germ cell cancer. *Cancer Invest.* **13 (4)**, 355–362.

SHEA TC, FLAHERTY M, ELIAS A, EDER JP, ANTMAN K, BEGG C, SCHNIPPER L, FREI E AND HENNER WD. (1989). A phase I clinical and pharmacokinetic study of carboplatin and autologous bone marrow support. *J. Clin. Oncol.*, **7**, 651–661.

SHEA TC, MASON JR, STORNIOLO AM, BISSENT E, BRESLIN M, MULLEN M AND TAETLE R. (1993). High-dose carboplatin chemotherapy with GM-CSF and peripheral blood progenitor cell support, a model for delivering repeated cycles of dose-intensive therapy. *Cancer Treat. Rev.*, **1**, 11–20.

SIEGERT W, BEYER J, STROHSCHEER I, BAURMANN H, OETTLE H, ZINGSEM J, ZIMMERMANN R, BOKEMEYER C, SCHMOLL HJ AND HUHN D. (1994). High-dose treatment with carboplatin, etoposide, and ifosfamide followed by autologous stem cell transplantation in relapsed or refractory germ cell cancer, a phase I/II study. *J. Clin. Oncol.*, **12**, 1223–1231.

SØRENSEN BT, STRÖMGREN A, JAKOBSEN P AND JAKOBSEN A. (1993). A limited sampling method for estimation of the carboplatin area under the curve. *Cancer Chemother. Pharmacol.*, **31**, 324–327.

VAN DER VIJGH WJF. (1991). Clinical pharmacokinetics of carboplatin. *Clin. Pharmacokinet.*, **21**, 242–261.

VAN DER WALL E, RICHEL DJ, HOLTKAMP MJ, SLAPER-CORTENBACH ICM, VAN DER SCHOOT CE, DALESIO O, NOOIJEN WJ, SCHORNAGEL JH AND RODENHUIS S. (1994). Bone marrow reconstitution after high-dose chemotherapy and autologous peripheral blood progenitor cell, effect of graft size. *Ann. Oncol.*, **5**, 795–802.

VAN DER WALL E, NOOIJEN WJ, BAARS JW, HOLTKAMP MJ, SCHORNAGEL JH, RICHEL DJ, RUTGERS EJT, SLAPER-CORTENBACH ICM, VAN DER SCHOOT CE AND RODENHUIS S. (1995). High-dose carboplatin, thiotepa and cyclophosphamide (CTC) with peripheral blood stem cell support in the adjuvant therapy of high-risk breast cancer, a practical approach. *Br. J. Cancer*, **71**, 857–862.

VAN WARMERDAM LJC, RODENHUIS S, VAN TELLINGEN O, MAAS RAA AND BEIJNEN JH. (1994*a*). Validation of a limited sampling model for carboplatin in a high dose chemotherapy combination. *Cancer Chemother. Pharmacol.*, **35**, 179–181.

VAN WARMERDAM LJC, TEN BOKKEL HUINICK WW, MAES RAA AND BEIJNEN JH. (1994*b*). Limited sampling models for anticancer agents. *J. Cancer Res. Clin. Oncol.*, **120**, 427–433.

VAN WARMERDAM LJC, VAN TELLINGEN O, MAES RAA AND BEIJNEN JH. (1995). A validated method for the analysis of carboplatin using Zeeman atomic absorption spectrometry. *Fres. J. Anal. Chem.*, **351**, 777–781.

VERMORKEN, J.B, TEN BOKKEL HUININK W.W, EISENHOWER EA, FAVALLI G, BELPOMME D, CONTE PF AND KAYE SB. (1993). Carboplatin versus cisplatin. *Ann. Oncol.*, **4** (suppl. 4), S41–S48.

WAGSTAFF AJ, WARD A, BENFIELD P AND HEEL RC. (1989). Carboplatin, a preliminary review of its pharmacodynamic and pharmacokinetic properties and therapeutic efficacy in the treatment of cancer. *Drugs*, **37**, 162–190.